# Effects of adding nano-emulsified plant oil and probiotics to drinking water during different periods besides sex on processing characteristics, physicochemical properties, and meat quality traits of broiler chickens

**DOI:** 10.3389/fvets.2023.1133605

**Published:** 2023-02-21

**Authors:** Gamaleldin M. Suliman, Elsayed O. S. Hussein, Ahmed Alsagan, Abdullah N. Al-Owaimer, Rashed Alhotan, Hani H. Al-Baadani, Hani A. Ba-Awadh, Mohammed M. Qaid, Ayman A. Swelum

**Affiliations:** ^1^Department of Animal Production, College of Food and Agriculture Sciences, King Saud University, Riyadh, Saudi Arabia; ^2^King Abdulaziz City for Science and Technology, Riyadh, Saudi Arabia

**Keywords:** broiler sex, processing performance, physicochemical properties, breast meat quality, nano-emulsified plant-oil, probiotics

## Abstract

**Introduction:**

High-quality meat is one of the consumer demands. Therefore, several studies have concluded that supplementing broilers with natural additives can improve meat quality. This study was carried out to evaluate the effects of nano-emulsified plant oil (Magic oil^®^) and probiotic (Albovit^®^) as water additives (at the rate of 1 ml/L and 0.1 g/L, respectively) during different growing periods on processing characteristics, physicochemical properties, and meat quality traits of broilers chickens.

**Methods:**

A total number of 432-day-old Ross broiler chicks were randomly assigned to one of six treatment groups according to the growing periods in which magic oil and probiotics were added to drinking water, each with nine replicates and eight birds per replicate (4♂ and 4♀). On day 35, birds' processing characteristics, physicochemical properties, and meat quality traits were examined.

**Results and discussion:**

The results showed that treatments had a significant (*P* < 0.001) impact on cooking loss, cohesiveness, and chewiness. The male broiler chickens had higher (*P* ≤ 0.05) initial lightness, initial whiteness index, water holding capacity, shear force, live weight, hot and chilled carcass weights, as well as lower gizzard and neck percentages than females. The interactions between treatments and sex showed a significant (*P* < 0.001) impact on cooking loss, shear force, hardness, springiness, and chewiness. In conclusion, supplementing male broiler chickens with Magic oil and probiotic, particularly from 0–30 days of age had favorable meat chewiness as a result of lower cohesiveness and hardness higher springiness, and the most convenient cooking loss value. Magic oil and probiotic, especially in males, is advisable to be supplemented in water of growing broilers chicken programs from 0 to 30 days of age. Moreover, further studies under commercial conditions are recommended to locate the most favorable combination of Magic oil/probiotic supplements for the best processing characteristics and meat quality attributes outcomes.

## 1. Introduction

Poultry production is presumably the most rapidly growing, adaptable, and profitable of all livestock sectors expanding in both developed and developing countries (1, 2). Poultry meat is widely accepted and consumed by people from all walks of life due to its high nutritional quality, delicious taste, low cost, and importance as an animal protein source in human growth and development (3–5). Continuous genetic selection has produced fast-growing broilers with a short production cycle that reach market weight at 6 weeks of age and have a high meat yield, accounting for 74.81% (1, 5–7). The selection has had a negative impact on meat quality by increasing fiber diameters and the ratio of glycolytic fibers (8). Meat quality is a multifaceted character that is influenced by breed, strain, age, genetic, environmental, sex, and nutritional factors (2, 9, 10). Several studies have been conducted to assess the impact of nutrient supplementation on meat quality in chickens (11–14).

Antimicrobial growth promoters (AGPs) have been used in the poultry production industry for decades, resulting in a high risk of antibiotic-resistant bacteria being transferred to humans. It is difficult to raise broiler chickens in an antibiotic-free production system, and finding an effective nutritional alternative to support growth performance, gut health, and functionality without using AGPs is critical (15). Several non-antibiotic growth promoters are commercially available to improve bird growth, control pathogens, and reduce the risks of antibiotic resistance and misuse (16, 17). Examples include herbal essential oils, extracts, nano-emulsions and secondary metabolites (18, 19). Additionally, exogenous enzymes (20), organic acids (21), probiotics (15, 22), prebiotics (23), amino acids (24, 25), and green nanoparticles (26).

Essential oils (EOs), a type of phytogenic, are viable substitutes for increasing meat broiler production efficiency (27). EOs can be used therapeutically in a variety of broiler production situations due to their antibacterial, antiviral, antifungal, and antiparasitic properties (18). A combination of canola oil nano-emulsion and 2% Satureja bachtiarica essential oil is an effective natural preservative for chilled chicken breast (28). Rabbits, monogastric, fed a diet containing nano-emulsified essential oil had a higher final live weight, a higher carcass weight, a higher meat protein content, a lower fat content, and higher monounsaturated and polyunsaturated acids (29). Probiotics in animal feed is projected to attain massive global growth, reaching USD 6.24 billion by 2026 (15). High dietary energy content increases carcass weight (CW), carcass yield, and abdominal fat (30). The quality of chicken meat is becoming a significant issue when viewed from the perspectives of consumers and industries (31). The criteria for meat quality include pH, color, water holding capacity, cook loss, myofibril fragmentation index, and shear force (2). Texture profile analysis is a constructive technique that uses a popular double compression test to mimic the bite action of the mouth to determine the textural properties of poultry meat (32).

There are very few studies in the literature that look at the effects of sex and water supplementation of nano-emulsified plant-oil on meat attributes *viz* processing characteristics, physicochemical properties, and meat quality traits of broiler chicken. On the other hand, the addition of nanoparticles such as zinc nanoparticles and curcumin nanoparticles to broiler feed and *Bacillus licheniformis* improved the weight, carcass characteristics, and meat quality of chickens (18). Thus, the objective of this study was to determine the effects of water supplements of Magic oil plus probiotics and sex on processing characteristics, physicochemical properties, and meat quality traits of broiler chickens.

## 2. Materials and methods

This study was approved by the Ethics Committee of Scientific Research, King Saud University (KSU), Saudi Arabia (Approval No: KSU-SE-21-02).

### 2.1. Nano-emulsified plant-oil and probiotics composition

#### 2.1.1. Magic oil ™ Atcopharma

Each liter contains 98.5% nano-emulsified crude oil including 26% monounsaturated fat, 59% polyunsaturated fat (50% linoleic acid; omega-6 & 7% linolenic acid; omega-3;), 14% saturated fats only and vitamin E.

#### 2.1.2. Albovit^®^ Albafarma

Each kg contains *Enterococcus faecium* (3.3X1012 CFU), Galacto-oligosaccharides (136,000 mg), Vitamin D3 (200,000 IU), and Vitamin C (200,000 mg).

### 2.2. Experimental design and bird's management

On arrival from Alkhumasia commercial hatchery, Riyadh, Saudi Arabia, 432-day-old broiler chicks (Ross 308) were sexed and individually weighed before being divided into six treatments based on body weights. Each group was further redistributed to nine replicates with eight birds per replicate (4♂ and 4♀). The study was carried out in an environmentally controlled poultry unit at temperatures 22–24°C. All stages of growing period were performed in the Animal Production Department, College of Food and Agriculture Sciences, King Saud University (24°43′28.8″N 46°37′07.9″E). Broiler chicks were raised in flour cage pens under similar managerial and hygienic conditions. A standard starter (0–15) and finisher diets (16–35 days) as shown in Table 1, isocaloric and isonitrogenous contents were offered in mash form based on corn-SBM and were formulated to meet or exceed the recommendations in commercial practice in Saudi Arabia. The two additives were supplemented in drinking water and were not included in the nutrient matrix. Upon arrival, the chicks were randomly distributed to one of six treatments according to the periods in which Magic oil and probiotics were added to the drinking water: Control, no additive (A), Magic oil and probiotics from days 1 to 35 (slaughter day) (B), Magic oil and probiotics from days 1 to 4 then from days 17 to 21, and from day 25 to slaughter (C), Magic oil and probiotics from days 1 to 4 then from day 7 to slaughter (D), Magic oil and probiotics from days 1 to 4 and from day 21 to slaughter (E) and probiotic from days 1 to 4 and from days 16 to 18 (P). Magic oil and probiotic were added to water at the rate of 1 ml/L and 0.1 g/L, respectively. Water (with or without Magic oil and probiotics) was available *ad libitum* for all birds in all groups.

**Table 1:** Dietary composition during starter and finisher periods.

**Ingredient %**	**Treatment period (0–49) days**	
	**Starter (0–21)**	**Finisher (22–49)**
Yellow corn	57.39	61.33
Soybean meal	27.00	22.80
Palm oil	2.20	2.80
Corn gluten meal	8.80	6.0
Wheat bran	0.00	3.0
DCP	2.30	2.09
Ground limestone	0.70	0.62
Choline chloride	0.05	0.05
DL-methionine	0.105	0.075
L-lysine	0.39	0.36
Salt	0.40	0.20
Threonine	0.17	0.17
V-M premix^a^	0.50	0.50
Total	100	100
**Analysis**		
ME, kcal/kg	3,000	3,050
Crude protein, %	23.0	20.5
Non phytate P, %	0.48	0.44
Calcium, %	0.96	0.88
Digestible lysine, %	1.28	1.15
Digestible methionine, %	0.60	0.54
Digestible sulfur amino acids, %	0.95	0.86
Digestible threonine, %	0.86	0.77

a Vitamin-mineral premix contains in the following per kg: vitamin A, 2,400,000 IU; vitamin D, 1,000,000 IU; vitamin E, 16,000 IU; vitamin K, 800 mg; vitamin B1, 600 mg; vitamin B2, 1,600 mg; vitamin B6, 1,000 mg; vitamin B12, 6 mg; niacin, 8,000 mg; folic acid, 400 mg; pantothenic acid, 3,000 mg; biotin 40 mg; antioxidant, 3,000 mg; cobalt, 80 mg; copper, 2,000 mg; iodine, 400; iron, 1,200 mg; manganese, 18,000 mg; selenium, 60 mg, and zinc, 14,000 mg.

### 2.3. Meat quality characteristics

#### 2.3.1. pH and temperature

The initial pH and temperature of the breast muscle were measured directly after slaughter (~15 min) and then 24 h later (ultimate) using a microprocessor pH- Meter (Model PH 211, Hanna Instruments). Two readings were taken, and the mean value was calculated for each carcass.

#### 2.3.2. Meat color

The color values of CIELAB Color System (1976), L^*^ (lightness) a^*^ (redness), and b^*^ (yellowness), were determined on the breast muscles 15 min after slaughter using a Chroma meter (Konica Minolta, CR-400-Japan). As described by Valizadeh et al. (33) and Qaid et al. (34), values for L^*^, a^*^, and b^*^ were converted to estimate the saturation index, total color change (ΔE), hue angle, browning index (BI), and whiteness index (WI). These parameters could provide more accurate assessment of how consumers perceive meat color.

#### 2.3.3. Water holding capacity

It was determined based on the technique described by Wilhelm et al. (35). Two replicates of around about 2 g were collected from the breast muscle of each sample and were cut into cubes. Then, the samples were placed between two filter papers and two Plexiglas and were left under a 10-kg weight for 5 min. Afterward, the samples were weighed and WHC was determined as the difference between the initial and final weights.

#### 2.3.4. Cooking loss

The frozen breast muscle (~100 g) was thawed overnight at 4°C. Then, they were placed in a commercial indoor countertop grill and were cooked to an internal temperature of 70°C. The temperature was monitored by inserting a thermocouple thermometer probe (Eco scan Temp JKT, Eutech Instruments) into the geometric center of the muscle. The muscles were weighed before and after cooking to determine cooking loss (CL) as the difference between the initial and final weights.

#### 2.3.5. Myofibril fragmentation index

As an indirect measure of calpain activity, myofibril fragmentation (MFI) in muscle samples was assessed. A total of 4 g of minced muscles, free of visible connective tissue and external fat, were homogenized for 30 s in a blender (Ultra Turrax; IKA-Werke, Staufen, Germany) with 40 ml of cold MFI buffer at 2°C. Following several washes, suspension aliquots were diluted in MFI buffer to a final protein concentration of 0.5 mg/ml and poured into a cuvette for immediate absorbance measurement at 540 nm with a spectrophotometer (HACH DR/3000 Spectrophotometer, USA). Each sample's MFI was multiplied by 200.

#### 2.3.6. Shear force

The cooked samples used in the cooking loss were reused for determining the shear force and were used to evaluate shear force according to Wheeler et al. (36). They were cooled to room temperature (21°C), then five 1.27 cm in diameter round cores were removed from each muscle sample parallel to the longitudinal orientation of the muscle fibers. Cores were obtained using a handheld coring device. Shear force was determined as the maximum force (*N*) perpendicular to the fibers using a Texture Analyzer (TA-HD-Stable MicroSystems, England) equipped with a Warner-Bratzler attachment. The crosshead speed was set at 200 mm/min.

#### 2.3.7. Texture profile analysis

The TPA parameters of hardness, chewiness, springiness, and cohesiveness were conducted and measured in the same manner as described in Qaid et al. (34) and Novaković and Tomašević (37).

### 2.4. Carcass measurements

At day 35 of age, 18 birds per treatment (nine males and nine females) were selected randomly. After slaughtering, feathers, heads, and shanks were removed and the remaining carcass was dissected to separate breast and thigh. Similarly, fat, liver, heart, gizzard, wings, and drumstick were also separated and weighed. The percentage of the yield of each part was calculated based on dressing weight (38).

### 2.5. Statistical analysis

The data were subjected two-way analysis of variance (ANOVA) in a general linear model (GLM) using Statistical Analysis System package (SAS) version 9.4 software (SAS Institute Inc., Cary, NC, USA) (39).

The model equation is described as follows:


$$\begin{array}{l} \gamma_{ij}=\mu +{T_{i}+G}_{j}+TG_{ij}\;+\;e_{ijk} \end{array}$$

Where:

Yij = the individual observation;
μ = the general experimental mean;
Ti = the effect of ith treatment;
Gj = the effect of *j*th sex;
TGij = the effect of treatment by sex interaction;
eijk = a random error.

Means for measurements showing significant differences in the analysis of variance were tested using the PDIFF option. The overall level of statistical significance was set at *P* ≤ 0.05. All values were expressed as statistical means ± standard error of the mean (SEM).

## 3. Results

The results of the physicochemical properties are shown in Tables 2–4. The results of the main effects: treatments and sex of broilers, and their interactions on the initial and ultimate color components (lightness, redness, and yellowness) and their derivatives of breast meat at 35 days of age are summarized in Tables 2, 3, respectively. While the results of the main effects; treatments and sex and their interactions on the initial and final values of pH and temperature of breast meat at 35 days of age are shown in Table 4. The results of meat quality characteristics of both male and female broiler chickens supplemented with Magic oil and probiotic are shown in Table 5. The data in Table 6 show the main effects of treatments and sex on carcass measurements at 35 days of age, as well as the effects of interaction between treatments and sex of broilers. The effects of treatments, sex, and their interaction on processing performance (weight of chilled carcass, yield of cooked carcass, breast, legs, wings, back, and neck in percent) at 35 days of age are shown in Table 7.

**Table 2:** Effects of adding nano-emulsified plant oil and probiotics to drinking water during different periods besides sex on initial color components and its derivative of breast meat of broiler chickens at 35 days of age.

**Sex**	**Treatment^a^**	***n*** ^b^	**Initial color components**			**Initial color derivatives**				
			Li*	ai*	bi*	Δ**E**	**Chroma**	**HA (**°**)**	**BI**	**WI**
**Interaction effects:**										
Males	A	9	48.29	3.73	4.24	46.12	5.73	46.85	14.74	47.95
	B	9	47.1	3.68	4.88	47.28	6.13	52.73	16.45	46.74
	C	9	46.3	3.9	4.29	48.10	5.92	47.31	15.66	45.97
	D	9	46.62	3.78	4.33	47.78	5.85	48.57	15.52	46.29
	E	9	47.48	4.19	4.98	46.97	6.60	49.42	17.26	47.05
	P	9	46.89	4.82	4.51	47.75	7.29	43.24	18.50	46.33
Females	A	9	43.67	4.98	4.47	50.86	6.92	42.27	18.98	43.22
	B	9	45.27	3.35	5.14	49.09	6.22	56.28	17.26	44.90
	C	9	45.87	4.34	5.03	48.60	6.81	49.07	18.48	45.43
	D	9	45.28	4.07	4.8	49.14	6.35	49.53	17.67	44.89
	E	9	44.52	3.94	4.87	49.89	6.38	49.01	17.81	44.13
	P	9	44.85	4.77	5.03	49.68	7.07	46.84	19.57	44.36
SEM			1.06	0.45	0.48	0.764	0.400	2.70	1.11	0.757
**Main effects:**										
Sex	Males	54	47.11^a^	4.02	4.89	47.33^b^	6.25	48.02	16.35	46.72^a^
	Females	54	44.91^b^	4.24	4.54	49.54^a^	6.62	48.83	18.29	44.49^b^
SEM			0.43	0.19	0.2	0.441	0.231	1.56	0.642	0.437
Treatment	A	18	45.98	4.35	4.36	48.49	6.32	44.56	16.86	45.59
	B	18	46.18	3.52	5.01	48.18	6.17	54.51	16.85	45.82
	C	18	46.09	4.12	4.66	48.35	6.36	48.19	17.07	45.70
	D	18	45.95	3.92	4.56	48.46	6.10	49.05	16.60	45.59
	E	18	46	4.07	4.92	48.43	6.49	49.22	17.54	45.59
	P	18	45.87	4.8	4.77	48.71	7.18	45.04	19.03	45.35
SEM			0.75	0.32	0.34	0.764	0.400	2.70	1.11	0.757
**Source of variation:**						***P*** **-values**				
Sex			< 0.001	0.39	0.21	0.001	0.26	0.71	0.04	0.001
Treatment			0.99	0.13	0.79	1.00	0.45	0.13	0.67	1.00
Sex × treatment			0.47	0.53	0.97	0.47	0.74	0.90	0.84	0.47

a A, unsupplemented control; B, Magic oil supplementation from 0 to 30 days of age; C, Magic oil supplementation from 1 to 4, 17 to 21, and 25 to 35 days of age; D, Magic oil supplementation from 1 to 4 and 17 to 35 days of age; E, Magic oil supplementation from 1 to 4 and 21 to 35 days of age; P, probiotic supplementation from 1 to 4 and 16 to 18 days of age.

b Number of replicate pens.

L^*^ lightness, a^*^ redness, and b^*^ yellowness; ΔE, total color change; Chroma, saturation index; HA, hue angle (°); BI, browning index; WI, whiteness index. Means within a column with no common superscript differ significantly (*P* ≤ 0.05). SEM, standard error of the mean.

**Table 3:** Effects of adding nano-emulsified plant oil and probiotics to drinking water during different periods besides sex on ultimate color components and its derivatives of breast meat of broiler chickens at 35 days of age.

**Sex**	**Treatment^a^**	***n*** **^b^**	**Ultimate color components**			**Ultimate color derivatives**				
			Lu*	au*	bu*	Δ**E**	**Chroma**	**HA (**°**)**	**BI**	**WI**
**Interaction effects:**										
Males	A	9	50.02	5.54	9.13	44.90	10.71	58.83	28.19	48.85
	B	9	50.27	5.86	9.3	44.75	11.07	57.75	29.00	49.00
	C	9	48.9	5.38	8.6	45.95	10.23	58.60	27.11	47.85
	D	9	47.27	5.48	8.7	47.64	10.51	57.49	28.59	46.15
	E	9	51.64	5.97	9.99	43.54	11.78	59.17	29.58	50.16
	P	9	48.62	6.87	9.09	46.56	11.78	54.42	31.00	47.25
Females	A	9	48.64	5.97	9.34	46.39	11.36	57.15	30.04	47.37
	B	9	48.59	5.15	10.73	46.50	12.00	64.05	32.60	47.16
	C	9	47.74	5.87	9.57	47.25	11.29	58.50	31.49	46.50
	D	9	48.99	6.16	9.11	46.04	11.15	56.42	29.46	47.74
	E	9	48	5.7	9.16	46.91	10.84	57.56	29.74	46.86
	P	9	50.19	5.26	10.14	44.84	11.59	62.60	29.96	48.83
SEM			1.20	0.70	0.64	0.85	0.49	2.24	1.46	0.84
**Main effects:**										
Sex	Males	54	49.45	5.85	9.14	45.56	11.01	57.71	28.91	48.21
	Females	54	48.69	5.69	9.67	46.32	11.37	59.38	30.55	47.41
SEM			0.49	0.29	0.26	0.49	0.28	1.29	0.84	0.48
Treatments	A	18	49.33	5.75	9.24	45.65	11.03	57.99	29.12	48.11
	B	18	49.43	5.5	10.01	45.63	11.54	60.90	30.80	48.08
	C	18	48.32	5.63	9.09	46.60	10.76	58.55	29.30	47.17
	D	18	48.13	5.82	8.9	46.84	10.83	56.96	29.03	46.95
	E	18	49.82	5.84	9.57	45.23	11.31	58.36	29.66	48.51
	P	18	49.41	6.07	9.62	45.70	11.69	58.51	30.48	48.04
SEM			0.85	0.5	0.45	0.85	0.49	2.24	1.46	0.84
**Source of variation:**						***P*** **-values**				
Sex			0.27	0.69	0.15	0.27	0.37	0.36	0.17	0.24
Treatments			0.66	0.98	0.55	0.74	0.70	0.89	0.93	0.75
Sex × treatment			0.2	0.55	0.56	0.23	0.69	0.44	0.77	0.23

a A, unsupplemented control; B, Magic oil supplementation from 0 to 30 days of age; C, Magic oil supplementation from 1 to 4, 17 to 21, and 25 to 35 days of age; D, Magic oil supplementation from 1 to 4 and 17 to 35 days of age; E, Magic oil supplementation from 1 to 4 and 21 to 35 days of age; P, probiotic supplementation from 1 to 4 and 16 to 18 days of age.

b Number of replicate pens.

Lu^*^ ultimate lightness, au^*^ ultimate redness, and bu^*^ ultimate yellowness; ΔE, total color change; Chroma, saturation index; HA, Hue angle (°); BI, browning index; WI, whiteness index. Means within a column with no common superscript differ significantly (*P* ≤ 0.05). SEM, standard error of the mean.

**Table 4:** Effects of adding nano-emulsified plant oil and probiotics to drinking water during different periods besides sex on breast meat pH and temperature of broiler chickens at 35 days of age.

**Sex**	**Treatment^a^**	***n*** **^b^**	**pH**		**Temperature (** **°** **C)**	
			**Initial**	**Ultimate**	**Initial**	**Ultimate**
**Interaction effects:**						
Males	A	9	6.06	5.93	27.78	13.41
	B	9	6.14	5.96	27.44	12.68
	C	9	6.10	5.99	27.32	12.82
	D	9	6.03	5.98	27.48	12.43
	E	9	6.14	5.87	27.30	12.81
	P	9	6.19	5.99	26.51	12.71
Females	A	9	6.14	5.97	27.68	12.46
	B	9	6.10	5.99	27.27	12.48
	C	9	6.06	5.95	27.37	12.30
	D	9	6.05	5.99	27.58	12.69
	E	9	6.07	5.99	27.26	12.32
	P	9	6.21	5.97	27.01	12.80
SEM			0.06	0.04	0.31	0.30
**Main effects:**						
Sex	Males	54	6.11	5.95	27.31	12.81
	Females	54	6.10	5.98	27.36	12.51
SEM			0.02	0.02	0.13	0.12
Treatment	A	18	6.10	5.95	27.73	12.94
	B	18	6.12	5.98	27.36	12.58
	C	18	6.08	5.97	27.24	12.56
	D	18	6.04	5.98	27.53	12.56
	E	18	6.10	5.93	27.28	12.57
	P	18	6.20	5.98	26.76	12.76
SEM			0.04	0.03	0.22	0.22
**Source of variation:**				***P*** **-values**		
Sex			0.85	0.35	0.77	0.09
Treatment			0.22	0.78	0.07	0.76
Sex × treatment			0.84	0.43	0.92	0.39

a A, unsupplemented control; B, Magic oil supplementation from 0 to 30 days of age; C, Magic oil supplementation from 1 to 4, 17 to 21, and 25 to 35 days of age; D, Magic oil supplementation from 1 to 4 and 17 to 35 days of age; E, Magic oil supplementation from 1 to 4 and 21 to 35 days of age; P, probiotic supplementation from 1 to 4 and 16 to 18 days of age.

b Number of replicate pens. SEM, standard error of the mean.

**Table 5:** Effects of adding nano-emulsified plant oil and probiotics to drinking water during different periods besides sex on meat quality of broiler chickens at 35 days of age.

**Sex**	**Treatment^a^**	***n*** **^b^**	**WHC%**	**CL%**	**MFI**	**SF (*N*)**	**Texture profile analysis**			
							**Hardness (** ***N*** **)**	**Springiness**	**Cohesiveness**	**Chewiness**
**Interaction effects:**										
Males	A	9	31.31^bc^	25.69^a^	85.28	6.57^a^	8.72^abc^	0.78^b^	0.41	2.90^abc^
	B	9	34.12^abc^	18.96^e^	80.47	5.24^c^	7.38^c^	0.86^a^	0.39	2.38^c^
	C	9	36.18^a^	22.80^b^	88.86	5.35^bc^	11.01^a^	0.77^b^	0.42	3.66^ab^
	D	9	33.03^ab^c	23.63^b^	85.48	5.49^bc^	9.29^abc^	0.79^b^	0.40	3.12^abc^
	E	9	32.96^ab^c	20.14^e^	72.72	5.66^bc^	9.56^abc^	0.77^b^	0.43	3.66^ab^
	P	9	34.49^ab^	20.78^cde^	84.62	5.68^bc^	10.58^a^	0.78^b^	0.45	3.67^ab^
Females	A	9	35.25^ab^	19.95^e^	75.35	4.97^c^	9.14^abc^	0.78^b^	0.37	2.54^bc^
	B	9	31.68^abc^	17.12^f^	88.49	5.88^ab^	10.52^a^	0.77^b^	0.40	3.74^ab^
	C	9	32.00^ab^c	21.88^bcd^	85.72	4.95^c^	8.65^bc^	0.77^b^	0.41	2.71^abc^
	D	9	31.25^bc^	22.52^bc^	82.45	4.87^c^	8.85^abc^	0.81^ab^	0.42	3.19^abc^
	E	9	32.45^abc^	22.75^b^	90.54	5.42^bc^	9.23^abc^	0.87^a^	0.44	3.54^ab^
	P	9	29.62^c^	22.72^bc^	89.60	5.54^bc^	10.72^a^	0.77^b^	0.45	3.95^a^
SEM			1.44	0.77	5.48	0.28	0.74	0.02	0.01	0.25
**Main effects:**										
Sex	Males	54	33.68	21.99	82.90	5.66^a^	9.42	0.79	0.42	3.23
	Females	54	32.04	21.16	88.59	5.27^b^	9.52	0.80	0.42	3.28
SEM			0.59	0.31	2.24	0.12	0.30	0.01	0.01	0.10
Treatment	A	18	33.28	22.82^a^	80.32	5.77	8.93	0.78	0.39^d^	2.72^c^
	B	18	32.90	18.04^b^	84.48	5.56	8.95	0.82	0.40^cd^	3.06^bc^
	C	18	34.09	22.34^a^	87.29	5.15	9.83	0.77	0.42^bc^	3.19^bc^
	D	18	32.14	23.07^a^	93.65	5.18	9.07	0.80	0.41^bcd^	3.16^bc^
	E	18	32.70	21.45^a^	81.63	5.54	9.40	0.82	0.43^ab^	3.60^ab^
	P	18	32.06	21.75^a^	87.11	5.61	10.05	0.77	0.45^a^	3.81^a^
SEM			1.02	0.54	3.88	0.20	0.52	0.02	0.01	0.18
**Source of variation**			***P*** **-values**							
Sex			0.05	0.06	0.08	0.02	0.82	0.72	0.91	0.76
Treatment			0.74	< 0.001	0.19	0.18	0.16	0.07	< 0.001	< 0.001
Treatment × sex			0.04	< 0.001	0.09	0.01	0.02	0.01	0.09	< 0.001

a A, un-supplemented control; B, Magic oil supplementation from 0 to 30 days of age; C, Magic oil supplementation from 1 to 4, 17 to 21, and 25 to 35 days of age; D, Magic oil supplementation from 1 to 4 and 17 to 35 days of age; E, Magic oil supplementation from 1 to 4 and 21 to 35 days of age; P, probiotic supplementation from 1 to 4 and 16 to 18 days of age. Means within a column with no common superscript differ significantly (*P* ≤ 0.05).

b Number of replicate samples.

**Table 6:** Effects of adding nano-emulsified plant oil and probiotics to drinking water during different periods besides sex on carcass measurements of broiler chickens at 35 days of age.

**Sex**	**Treatment^a^**	***n*** **^b^**	**Live weight (g)**	**Hot carcass weight (g)**	**Carcass yield (%)**	**Abdominal fat (%)**	**Liver (%)**	**Heart (%)**	**Gizzard (%)**
**Interaction effects:**									
Males	A	9	2,590.6	1,898.4	73.3	1.3	2.2	0.5	1.5
	B	9	2,683.3	1,977.8	73.7	1.3	2.2	0.5	1.4
	C	9	2,546.7	1,857.9	72.9	1.3	2.4	0.5	1.5
	D	9	2,507.8	1,859.9	74.2	1.3	2.2	0.6	1.5
	E	9	2,658.9	1,961.3	73.8	1.4	2.3	0.6	1.4
	P	9	2,564.4	1,870.0	73.0	1.3	2.3	0.5	1.5
Females	A	9	2,103.3	1,529.3	72.7	1.2	2.3	0.5	1.6
	B	9	2,235.0	1,642.1	73.4	1.5	2.3	0.6	1.5
	C	9	2,150.0	1,570.0	73.0	1.4	2.4	0.5	1.6
	D	9	2,269.4	1,643.1	72.4	1.3	2.4	0.6	1.5
	E	9	2,169.4	1,601.1	73.8	1.3	2.3	0.5	1.6
	P	9	2,245.6	1,646.6	73.3	1.5	2.3	0.6	1.5
SEM			61.4	48.2	0.5	0.1	0.1	0.0	0.1
**Main effects:**									
Sex	Males	54	2,591.9^a^	1,904.2^a^	73.5	1.3	2.3	0.5	1.5^b^
	Females	54	2,195.5^b^	1,605.4^b^	73.1	1.4	2.3	0.5	1.6^a^
SEM			25.1	19.7	0.2	0.1	0.0	0.0	0.0
Treatment	A	18	2,346.9	1,713.9	73.0	1.3	2.2	0.5	1.5
	B	18	2,459.2	1,809.3	73.5	1.4	2.3	0.5	1.5
	C	18	2,348.3	1,713.9	73.0	1.4	2.4	0.5	1.5
	D	18	2,388.6	1,751.5	73.3	1.3	2.3	0.6	1.5
	E	18	2,414.2	1,781.2	73.8	1.4	2.3	0.5	1.5
	P	18	2,405.0	1,758.3	73.1	1.4	2.3	0.5	1.5
SEM			43.4	34.1	0.4	0.1	0.1	0.0	0.1
**Source of variation:**					***P*** **-values**				
Sex			< 0.001	< 0.001	0.24	0.47	0.39	0.49	0.02
Treatment			0.44	0.30	0.60	0.91	0.66	0.49	0.96
Sex × treatment			0.25	0.44	0.37	0.54	0.89	0.20	0.93

a A, unsupplemented control; B, Magic oil supplementation from 0 to 30 days of age; C, Magic oil supplementation from 1 to 4, 17 to 21, and 25 to 35 days of age; D, Magic oil supplementation from 1 to 4 and 17 to 35 days of age; E, gic oil supplementation from 1 to 4 and 21 to 35 days of age; P, probiotic supplementation from 1 to 4 and 16 to 18 days of age.

b Number of replicate samples. The relative weights of abdominal fat, liver, heart, and gizzard were calculated relative to live weight. Means within a column with no common superscript differ significantly (*P* ≤ 0.05). SEM, standard error of the mean.

**Table 7:** Effects of adding nano-emulsified plant oil and probiotics to drinking water during different periods besides sex on processing performance of broiler chickens at 35 days of age.

**Sex**	**Treatment^a^**	***n*** **^b^**	**Chilled carcass weight (g)**	**Carcass yield (%)**	**Breast (%)**	**Legs (%)**	**Wings (%)**	**Back (%)**	**Neck (%)**
**Interaction effects:**									
Males	A	9	1,871.6	72.3	26.4	19.7	6.6	13.1	4.9
	B	9	1,944.4	72.4	28.1	19.3	6.4	12.9	4.2
	C	9	1,832.2	71.9	26.9	19.6	6.5	13.3	3.9
	D	9	1,759.1	70.5	26.9	19.2	6.5	12.6	3.7
	E	9	1,932.7	72.7	27.0	20.2	6.5	13.2	4.2
	P	9	1,834.4	71.5	27.1	19.6	6.4	12.9	4.1
Females	A	9	1,504.5	71.5	27.0	18.9	6.8	12.8	4.5
	B	9	1,606.4	71.8	26.4	19.0	6.7	13.2	4.7
	C	9	1,617.8	75.6	29.3	19.4	6.8	13.4	5.2
	D	9	1,618.9	71.3	26.5	19.1	6.6	13.2	4.4
	E	9	1,573.6	72.5	27.8	17.2	6.9	13.6	5.0
	P	9	1,627.3	72.5	26.9	19.4	6.6	13.2	5.0
SEM			50.4	1.4	0.8	0.7	0.2	0.4	0.3
**Main effects:**									
Sex	Males	54	1,862.4^a^	71.9	27.1	19.6	6.5^b^	13.0	4.2^b^
	Females	54	1,591.3^b^	72.5	27.3	18.8	6.7^a^	13.3	4.8^a^
SEM			20.6	0.6	0.3	0.3	0.1	0.2	0.1
Treatment	A	18	1,687.8	71.9	26.7	19.3	6.7	12.9	4.7
	B	18	1,775.4	72.1	27.3	19.2	6.6	13.1	4.5
	C	18	1,725.0	73.8	28.1	19.5	6.6	13.3	4.5
	D	18	1,689.0	70.9	26.7	19.1	6.6	12.9	4.1
	E	18	1,753.1	72.6	27.4	18.7	6.7	13.4	4.6
	P	18	1,730.9	72.0	27.0	19.5	6.5	13.1	4.5
SEM			35.6	1.0	0.6	0.5	0.1	0.3	0.2
**Source of variation:**					***P*** **-values**				
Sex			< 0.001	0.43	0.59	0.07	0.008	0.27	0.002
Treatment			0.45	0.50	0.57	0.86	0.80	0.72	0.52
Sex × treatment			0.12	0.64	0.26	0.28	0.94	0.92	0.21

a A, unsupplemented control; B, Magic oil supplementation from 0 to 30 days of age; C, Magic oil supplementation from 1 to 4, 17 to 21, and 25 to 35 days of age; D, Magic oil supplementation from 1 to 4 and 17 to 35 days of age; E, Magic oil supplementation from 1 to 4 and 21 to 35 days of age; P, probiotic supplementation from 1 to 4 and 16 to 18 days of age.

b Number of replicate pens. Means within a column with no common superscript differ significantly (*P* ≤ 0.05). SEM, standard error of the mean.

### 3.1. Effects of treatments

Data analysis revealed that water supplementation of Magic oil and probiotic had no significant effect on the initial and final color components of breast flesh (Tables 2, 3). The supplementation also had no significant effect on the initial and final values of pH and temperature of breast meat at 35 days of age, as shown in Table 4. Table 5 shows that treatments had no effect on WHC, MFI, SF, hardness, and springiness but had an effect on CL, cohesiveness, and chewiness. Compared to the other groups, Magic oil supplementation had the lowest (most convenient) value (18.04) for cooking loss at 0–30 days of age. The most convenient values for cohesiveness and chewiness were obtained by the control group. In contrast, probiotic supplementation at 0–4 and 16–18 days of age resulted in the highest values for cohesiveness and chewiness. In Magic oil groups, the values were intermediate. The experimental treatments had no effect (*P* ≥ 0.05) on carcass measurements and processing performance at 35 days of age (Tables 6, 7).

### 3.2. Effects of sex

Statistical analysis of the data revealed that sex had no significant effect (*P* > 0.05) on the initial and final color components of breast meat (Tables 2, 3) and on the initial and final pH and temperature values of breast meat at 35 days of age (Table 4). However, the initial lightness index (*P* < 0.001) and initial whiteness index (*P* = 0.001) were influenced by sex, with male chickens having a higher value than female chickens. As shown in Table 5, the results of CL%, MFI%, and texture profile analysis were not significantly different in male and female broiler chickens (*P* > 0.05). However, the results showed that sex had a significant effect (*P* = 0.02) on SF and tended to be significant on the WHC ratio (*P* = 0.05), with male chickens having a higher value than female chickens (Table 5).

The effects of sex on carcass measurements of birds at 35 days of age are shown in Table 6. Male chickens had significantly (*P* ≤ 0.05) higher LW (2,591.9) and CW (1,904.2) at marketing age than females, which had LW and CW of 2,195.5 and 1,605.4, respectively. On the other hand, females had significantly higher gizzard weight (1.6%) than males (1.5%). The percentage of carcass yield, abdominal fat, liver, and heart did not differ significantly (*P* > 0.05) between male and female birds. At 35 days of age, sex had a significant effect on chilled carcass weight, wings, and neck percentage (Table 7). Males had a higher chilled carcass weight, lower relative wing, and neck weight than females. On the other hand, there were no significant (*P* > 0.05) differences between males and females in other processing performances (carcass yield, breast, legs, back, and neck percentages), as shown in Table 7.

### 3.3. Interaction of treatment and sex

Statistical analysis of the data revealed that the interaction of supplements and sex had no significant (*P* > 0.05) effect on the initial and final color components of breast flesh (Tables 2, 3). On the other hand, the interaction of supplements and sex shown in Table 4 had no significant effects (*P* > 0.05) on the initial and final values of pH and temperature of breast meat at 35 days of age.

In Table 5, the results show that the interaction of treatments and sex had a significant effect (*P* ≤ 0.05) on WHC, CL, SF, and texture profile analysis, except for cohesiveness. Males in the treatment group fed Magic oil from 0 to 4, 17 to 21, and 25 to 35 days of age, achieved the best WHC ratio (36.18), followed by females in the control group (35.25), and males in the treatment group (34.12) fed Magic oil from 0 to 30 days of age. In contrast, females in the treatment group fed probiotic at 1–4 and 16–18 days of age had the lowest WHC ratio values (29.62). Male and female birds in the treatment group receiving Magic oil supplementation from 0 to 30 days of age had the best CL ratios (18.96 and 17.12, respectively), whereas male birds in the control group had the worst (25.69). The female fed the Magic oil from 1 to 4 and 17 to 35 days of age had the lowest shear forces (4.87 *N*), indicating the greatest tenderness, and males fed the Magic oil from 0 to 30 days of age had the highest tenderness (5.24 *N*). The highest value of shear force in the males of the control group (6.57 *N*) indicated tough meat. The males fed Magic oil from 0 to 4, 17 to 21, and 25 to 35 days of age had the highest hardness (11.01 *N*), while the females fed Magic oil from 0 to 30 days of age had the lowest hardness (7.38 *N*). Compared to the other groups, female broilers supplemented with Magic oil at 1–4 and 21–35 days of age and male broilers supplemented with Magic oil at 0–30 days of age had the highest springiness values (0.87 and 0.86, respectively). Male broilers fed Magic oil at 0–30 days of age had the lowest values for chewiness (2.38), whereas female broilers fed probiotic at 1–4 and 16–18 days of age had the highest values for chewiness (3.95). Taken together, male broilers supplemented with Magic oil at 0–30 days of age had the best options in terms of chewiness (2.38) because they had lower cohesiveness (0.39) and hardness (7.38) and higher springiness (86), as well as the most favorable cooking loss value and water holding capacity.

The interaction of supplements and sex had no effect (*P* > 0.05) on carcass measurements and processing performance at 35 days of age (Tables 6, 7).

## 4. Discussion

The efficacy of Magic oil, a natural nano-emulsified plant-oil, was compared to probiotic on carcass traits and breast quality in broiler chickens in this study. Nano emulsions are used in the food industry to encapsulate, protect, deliver, and transport hydrophobic (low water solubility) bioactive components such as nutrients, nutraceuticals, antimicrobials, and antioxidants (40, 41). They are composed of tiny oil droplets suspended in water and act as a vehicle for essential oils to be bioavailable (18). Recent studies on the quality of meat or carcass traits of birds supplemented with powder, essential oils, or extracts of phytogenic in diets have been conducted (42–46). Unfortunately, little or no research has been conducted to examine the effect of water supplementation of Magic oil as nano-emulsified plant-oil and probiotic on the meat attributes of birds. At a probability level of α ≤ 0.05, the null hypothesis states that the effects of Magic oil and probiotic, sex, and their interactions on processing characteristics, physicochemical properties, and meat quality traits of broiler chickens are equal to the effects of the control group. Treatments, sex, and their interactions, according to the alternative hypothesis, improved some or all of the selected parameters.

Meat color is influenced by many factors, such as pre-slaughter factors, heme pigments, stunning methods, moisture content, cooling regimes, sex, strain, stress, and protein physical status (47). In agreement with Yetişir et al. (48), who noted that a higher L^*^ value would be preferable in terms of consumer acceptance. In our study, male birds had significantly higher initial L^*^ and whiteness index values (47.11 and 46.72, respectively) than females (44.91 and 44.49, respectively). Identifying color is an easy way to determine the pH of meat. If the meat is very dark, the pH is high, and if it is very light, the pH is low. Female birds in the control group, for example, were very light (Li^*^ = 48.29) and had a low pH (initial pH = 6.06). Birds given Magic oil between the ages of 0 and 30 days had a numerically higher initial Li^*^ = 46.18, resulting in a numerically higher initial WI = 45.82. Treatments, sex, or their interactions had no effect on the other color components and derivatives in breast meat. According to Abudabos et al. (49), no treatment effect of nano-emulsified plant oil or betaine was observed in L^*^, a^*^, b^*^, color saturation, hue angle (H°), and a^*^ to b^*^ ratio.

The pH is defined as the negative log of the concentration of hydrogen ions and the pH parameter was a good predictor of meat characters (9). After slaughter, oxygen deprivation raises hydrogen ion concentrations due to lactic acid dissociation *via* the anaerobic glycolysis pathway, resulting in a pH drop. The pH declines directly affect the protein solubility, protein denaturation, protein's capacity bind water, and shelf life (50–52). Thus, broiler breast meat with a high pH has a higher water-binding capacity than meat with a lower pH. Lower pH in bird meat groups with essential oil or phytogenic nano-emulsions essential oil may be responsible for inhibiting the integration of the deterioration of microorganism growth (53, 54). High pH (over 6.2) and low pH values (below 5.8) can negatively influence meat quality of broiler breast meat [dark, firm, and dry (DFD) vs. pale, soft, exudative (PSE), respectively] (46, 50, 55). As a result, our data fell within the normal meat quality pH values (5.9–6.2), with initial pH ranging from 6.03 to 6.21 and final pH ranging from 5.87 to 5.99. Protein denaturation, a protein's ability to bind water, tenderness, and springiness were not impacted by treatment because the concentration of hydrogen ions in the muscle of broiler breasts was unaffected by treatment. This contradicts Abudabos et al. (49), who stated that diet only affected breast filets pH at 24 h post-mortem; the control had lower breast pH than betaine, while nano-emulsified plant oil had an intermediate temperature.

The term “water holding capacity” describes a muscle's capacity to bind water under specific circumstances. Usually, a sharp pH drop in meat can denaturize proteins, leaving behind pale meat with low WHC. According to Hughes et al. (56), decreasing water retention is linked to a decrease in the nutritional value of the meat due to the loss of some nutrients, which makes the breast meat less tender. It also tends to result in less reflective surface light, which lowers L^*^ values. Cooking loss is the proportion of water lost during cooking due to shrinkage, which is related to the loss of juiciness to the palate. Usually, an increase in WHC accompanied by a decrease in the percentage CL (57, 58). Although cooking loss was significantly lower when broilers were supplemented with Magic oil from 0 to 30 days of age in both males and females, WHC in this study did not show any significant differences in treated groups compared with control.

Myofibrillar fragmentation is the extent of myofibrillar destruction caused by homogenization. The treatments had no effect on the MFI of the breast muscle. Myofibrillar fragmentation index values, according to Olson and Stromer (59), are strongly correlated with other muscle measurements such as tenderness. Magic oil supplementation, on the other hand, did not differ from the control in terms of myofibril fragmentation. The SF of the birds' breast muscle, on the other hand, ranged from 5.5 to 5.8 kgf/g (60) and from 2.71 to 3.31 kgf/g (61). As the SF values in this trial ranged between 5.15 and 5.77 *N*, the Magic oil or probiotic supplementation groups had no effect on meat tenderness. These findings are consistent with those of Pokoo-Aikins et al. (46), who found that different levels of dietary DL-Methionine supplementation had no effect on the meat toughness value of broilers. The dietary methionine level, the sex of the bird, and their interactions had no effect on the textural properties of cooked meat (46). According to Hussein et al. (10), females had more tender pectoral muscles and more myofibrillar fragmentation than males.

The texture profiles (cohesiveness and chewiness) of the treatments differed significantly, with the Magic oil groups having lower levels of cohesiveness and chewiness than the probiotic group. In general, the effect of treatment and sex interaction on meat tenderness resulted in variation between sexes rather than between treatment groups; thus, female meat was tenderer than male meat in each treatment group except in the group supplemented with Magic oil from 0 to 30 days of age, where male meat was tenderer than female. Furthermore, male meat in the control group was the highest SF value compared to other groups. Thus, meat quality could be enhanced by adding natural antioxidant compounds, and Magic oil has the highest antioxidant capacity due to its high phenolic content. Foods fortified with micro/nano encapsulated vegetable-essential oils can improve their functional properties such as antioxidant and antimicrobial activity, as well as having more healthy unsaturated fatty acids (62).

The current study found that Magic oil additives improve meat quality, especially when supplemented from 0 to 30 days of age, with favorable chewiness in female broiler chickens (2.38) that resulted in lower cohesiveness (0.39) and hardness (7.38) and higher springiness (86). Female cage-reared broilers had higher meat quality in the breast muscle (63). Due to chewiness equal cohesiveness^*^hardness^*^springiness, which indicated chewiness was influenced by one or more of these parameters and has a direct relationship. In addition, the group that supplemented with Magic oil from 0 to 30 days of age were the most convenient cooking loss value and holding water.

Female broiler chickens in the supplemented probiotic group from 0 to 4 and 16 to 18 days of age had a rapid drop in meat pH (0.24) within 24 h postmortem compared to the other group, resulting in low WHC (29.62). Furthermore, the probiotic group had the highest cohesiveness (0.45), greater hardness (10.72), and the lowest springiness (0.77), which resulted in higher chewiness (3.95) and lower tenderness (5.54). These findings support the findings of Loddi et al. (64) who noticed that probiotics added to water and feed had no effect on the sensory characteristics of meat. On the other hand, Jensen and Jensen (65) found a positive impact of probiotics including *Bacillus licheniformis* and *Bacillus subtilis* spores on the flavor of broiler meat after cooling for 5 days. Several studies have suggested that nanoemulsion-based products can positively influence the physicochemical and sensory properties of breast muscles (66). Conversely, some authors have observed that natural antioxidants have little or no effect on the sensory characteristics of meat. For example, dietary supplementation with nano-emulsified vegetable-essential oils (49) or probiotic (16) had no effect on the quality of chicken meat in terms of myofibril fragmentation index, cooking loss, shear force, and texture profile analysis.

In the current study, male birds had 18.06% more live weight, 18.61% more hot carcass weight, and 17.04% more chilled carcass weight, resulting in 0.55% more carcass yield than female birds at marketing age. Female birds, on the other hand, weighed 6.67% more relative gizzards, 14.29% more relative neck weight, and 3.08% more relative wings than males. These results agree with those reported by others (10, 30, 67). The percentages of carcass yield, abdominal fat, liver, and heart were not significantly different between males and females. These findings partially contradict Majid et al. (68), who found a significant difference between males and females in most carcass cut weights.

In this experiment, the same level of Magic oil was used at different stages of bird development: from day one to slaughter, from 1 to 4 days, then 17 to 21 days and 25 days, and from 1 to 4 days, then 7 days to slaughter. However, the obtained results in this study varied depending on the treatment period. As a result, more researches are prompted to investigate different levels of Magic oil at different growth periods of birds in order to determine the best level of supplementation. These findings are in contrast with Abudabos et al. (49) who stated that dietary supplements of nano-emulsified plant oil/betaine had no effect on dressing, leg, fat, gizzard, spleen, or thymus, but had a significant effect on breast meat, liver, and bursa and in contrast to Nisar et al. (16), who claimed that treatments had no effect on carcass characteristics.

## 5. Conclusions

In conclusion, both water supplements of Magic oil and probiotic had no negative effects on the color, pH, temperature, or processing performance of breast meat. Male broiler chickens supplemented with Magic oil from 0 to 30 days of age had the best options in terms of chewiness as they had lower cohesiveness and hardness besides higher springiness, as well as the most convenient cooking loss value and water holding capacity. The results showed that male birds had higher initial lightness, water holding capacity, shear force, live weight, hot and chilled carcass weights, as well as a lower gizzard and neck percentage than female. These findings could also be used as a foundation for future studies of water supplementation of nanoemulsified plant oil and probiotics and their effects on performance, carcass quality and meat traits of broiler chickens.

## Data availability statement

The raw data supporting the conclusions of this article will be made available by the authors, without undue reservation.

## Ethics statement

The animal study was reviewed and approved by the Ethics Committee of Scientific Research, King Saud University (KSU), Saudi Arabia (Approval No. KSU-SE-21-02).

## Author contributions

GS and EH: conceptualization. AA, GS, and EH: methodology and investigation. HA-B: software. RA, GS, and EH: validation. RA: formal analysis. AA-O: resources and funding acquisition. HB-A: data curation. MQ, GS, and EH: writing—original draft preparation. AS: writing—review and editing and supervision. GS: visualization. GS and AA-O: project administration. All authors have read and agreed to the published version of the manuscript. All authors contributed to the article and approved the submitted version.
